# Possible Mechanisms Involved in the Vasorelaxant Effect Produced by Anorexigenic Drugs in Rat Aortic Rings

**DOI:** 10.3390/medsci7030039

**Published:** 2019-02-27

**Authors:** Daniela García-Alonso, Dan Morgenstern-Kaplan, Ariel Cohen-Welch, Jair Lozano-Cuenca, Jorge Skiold López-Canales

**Affiliations:** 1 Centro de Investigación en Ciencias de la Salud Anáhuac (CICSA), Universidad Anáhuac México Campus Norte, Mexico City 52786, Mexico; danielaga_14@hotmail.com (D.G.-A.); dmorgensternk@gmail.com (D.M.-K.); ariel.cohen94@hotmail.com (A.C.-W.); 2 Department of Physiology and Cellular Development, National Institute of Perinatology, Mexico City 11000, Mexico; prayax@hotmail.com

**Keywords:** anorexigenics, obesity therapy, weight reduction, vasodilation, clobenzorex, fenproporex, amfepramone, triiodothyronine (T_3_)

## Abstract

Anorexigenics are compounds capable of reducing or suppressing appetite. Their three main types act on different neurotransmitters, either norepinephrine, serotonin or a combination of both. Among the drugs that act on norepinephrine are fenproporex, amfepramone and clobenzorex. Derivatives of the thyroid hormone triiodothyronine have also been associated with weight loss and used as a controversial treatment for obesity, despite their known cardiovascular side effects. Recent data suggest a possible vasodilating effect for these four substances that might be beneficial in a subset of patients. Herein we performed a systematic review of the literature (with emphasis on recent reports) to determine the implications and mechanisms of the vasodilating effects of some anorectics, specifically fenproporex, clobenzorex, amfepramone and triiodothyronine. Data analysis showed these four drugs to be vasodilating agents for rat aortic rings. The different mechanisms of action include endothelium-dependent vasodilation via activation of the NO-cGMP-PKG pathway and the opening of calcium-activated potassium channels. The finding of vasodilating activity indicates a potential role for some anorexigenic drugs in the treatment of obesity in hypertensive patients. Further in vivo studies are needed to test the clinical benefits of these four drugs.

## 1. Introduction

Nowadays, obesity represents a very serious public health problem, the prevalence of which is considered a pandemic of the 21st century. It is defined as a systemic, chronic and metabolic disorder associated with cardiovascular disease, diabetes, hypertension, dyslipidemia and a diminished life expectancy [1,2]. 

Among the multiple treatments available to manage obesity, lifestyle changes and exercise are considered the cornerstone. Nevertheless, many obese and overweight patients also benefit from pharmacological therapy. Indeed, the clinical guidelines of the Endocrine Society recommend the inclusion of diet and exercise in all regimens focused on weight loss, as well as pharmacotherapy for patients with a body mass index (BMI) ≥27 in case of presenting any comorbidity, and all of those with a BMI > 30 [3]. Consequently, there are a wide variety of drugs currently available for treating obesity, such as pancreatic lipase inhibitors, thermogenic agents and hunger suppressors [2,4]. Regarding appetite suppressors, some anorexigenic drugs serve this function. The three main types of anorexigenics act on different neurotransmitters, either norepinephrine, serotonin or a combination of both. 

Noradrenergic anorexigenic drugs are derived from amphetamines, which decrease or suppress appetite by increasing the release of catecholamines in the paraventricular nucleus of the hypothalamus. Amphetamines were withdrawn from the market because of being potentially addictive. New modified drugs have been developed with less risk of addiction, such as clobenzorex, fenproporex, mazindol, amfepramone and phentermine [5,6,7,8]. Nowadays, the latter is one of the most frequently used drugs for the management of obesity. Although phentermine was previously thought to have an addictive effect with long-term therapy; this was recently proven to be a misunderstanding [9,10,11,12].

Serotoninergic drugs are classified either as agonists or selective serotonin reuptake inhibitors (SRIs). Whereas the former trigger the release serotonin by its receptor, SRIs (e.g., fluoxetine and paroxetine) augment the extracellular concentration of this neurotransmitter by inhibiting its reuptake. The latter, usually prescribed for depression and other psychiatric disorders, are also helpful for promoting weight loss in the short term [5,7,8].

Finally, among the noradrenergic-serotoninergic drugs is a tertiary amine known as sibutramine. By acting through active metabolites that bind to the adrenergic α1, β1 and serotoninergic 2a and 2c receptors at the central level, it favors early satiety, stimulates thermogenesis and boosts energy expenditure, while showing low addictive capacity [5,7,13].

Another drug administered for the treatment of overweight and obesity is orlistat, an inhibitor of pancreatic lipases. Until 2012, it was the only anti-obesity drug to be approved for long-term use. This drug should be complemented with multivitamins, since it can reduce the absorption of liposoluble vitamins [14]. One study demonstrated beneficial results from the long-term intake of orlistat together with modifications in lifestyle (diet and physical activity). Over a one-year period, this combination led to significant weight loss (compared to the placebo) [15]. Recently, triiodothyronine (T_3_) or thyroid hormone derivatives have been sold and consumed illicitly to achieve weight loss, producing some cases of thyrotoxicosis or serious cardiac problems [14].

The following is a systematic review of the effects of a subgroup of anorexigenic drugs (e.g., amfepramone, T_3_, fenproporex and clobenzorex), including their vasodilator effect on rat aortic rings pre-contracted with phenylephrine.

## 2. Clobenzorex

Clobenzorex is well absorbed by the digestive tract. Its metabolism affords conjugated derivatives of clobenzorex, parahydroxyamphetamine, amphetamine and hippuric acid. Excreted in bile as conjugates of clobenzorex and parahydroxyamphetamine, it undergoes a more or less partial enterohepatic cycle. The fecal elimination of clobenzorex consists of unchanged products, conjugated derivatives, urinary parahydroxyclobenzorex (1.5–6% of the administered dose) and amphetamine (15%).

Among the adverse effects of clobenzorex are the consequences of vasoconstriction, including arterial hypertension and pulmonary artery hypertension. Vasoconstriction results from the increased flow of norepinephrine in the synaptic vesicles of sympathetic neurons, giving rise to the interaction of this neurotransmitter with α-1 receptors. Apart from hypertension, severe adverse events include polyuria, severe headaches and palpitations. Moderate side effects consist of depression, diarrhea and vomiting, while mild effects are irritability, dysuria, dry mouth and temporary anxiety [2,11,16,17,18,19].

A recent study by López et al. [17] showed that the acute application of clobenzorex induced an immediate concentration-dependent vasorelaxant effect on endothelium-intact but not on endothelium-denuded phenylephrine-precontracted rat aortic rings. The vasorelaxant effect produced by clobenzorex was significantly attenuated by L-NAME (NG-nitro-l-arginine methyl ester a direct inhibitor of nitric oxide synthase), ODQ (an inhibitor of nitric oxide-sensitive guanylyl cyclase), and KT 5823 (an inhibitor of protein kinase G). Hence, the stimulation of the NO-cGMP-PKG pathway is implied in the appetite suppressant effect. Whereas the vasorelaxant effect elicited by clobenzorex was unaffected by glibenclamide (an ATP-sensitive K^+^ channel blocker) and 4-Aminopyridine (4-AP, a voltage-activated K^+^ channel blocker), it was significantly attenuated by tetraethylammonium (TEA, a Ca^2+^-activated K^+^ channel blocker and non-specific voltage-activated K^+^ channel blocker) and apamin plus charybdotoxin (blockers of small- and large-conductance Ca^2+^-activated K^+^ channels, respectively). This points to the involvement of Ca^2+^-activated K^+^ channels in the effect. Furthermore, the vasorelaxation produced by clobenzorex was unaffected by indomethacin (a prostaglandin synthesis inhibitor), clotrimazole (a cytochrome P450 inhibitor) and cycloheximide (a general protein synthesis inhibitor), thus excluding the participation of prostacyclins, endothelial-derived hyperpolarizing factor (EDHF) or protein synthesis in the endothelium-mediated vasorelaxation. Overall, these findings suggest that the NO/cGMP/PKG/Ca^2+^-activated K^+^ channel pathway is a plausible mechanism for the observed vasorelaxant effect [17].

## 3. Fenproporex

Another anorectic is fenproporex, chemically denoted as (±) N-2-cyanoethylamphetamine or (±) 3-[(1-methyl-2-phenethyl)amino]propionitrile. Since this drug has exhibited lipolytic action as well as effects in the hypothalamic nuclei that suppress appetite, it is used in the treatment of obesity and overweight. 

Fenproporex is metabolized to amphetamine, which is a stimulator of the central and peripheral nervous system. In vivo studies on humans have shown two partially overlapping metabolic pathways for fenproporex: ring alteration by single and double aromatic hydroxylation followed by methylation and side chain degradation to amphetamine by *N*-dealkylation. The possible involvement of CYP2D6 in the formation of amphetamine from such precursor drugs is still controversial. Some authors have tried to explain the large differences in the amount of amphetamine metabolically formed from each of these precursors in function of CYP2D6 polymorphism [20]. 

The maximal plasmatic concentration of fenproporex occurs 2–4 h post-administration and its effect lasts for 6–8 h. It has renal clearance and eliminates fully after 48 h [2,7,21,22]. Among the adverse events are glaucoma, pulmonary artery hypertension, vomiting, headaches, vertigo, tachycardia and insomnia [19]. It is contraindicated in patients suffering from psychiatric disorders, drug abuse, alcoholism, hypertension, coronary artery disease and pulmonary hypertension [7,22,23].

According to recent reports, fenproporex induces a concentration-dependent relaxation on phenylephrine-precontracted rat aortic rings with functional endothelium, suggesting that its vasorelaxant effects are endothelium-dependent. The finding of a vasorelaxant effect elicited by fenproporex diverges from the vasoconstrictor effect of antiobesity drugs found in several in vitro and in vivo studies. Moreover, such a vasorelaxant response contrasts with the finding of increased plasma serotonin levels stemming from the intake of this drug, possibly implicating fenproporex in the development of acute cardiac and pulmonary disorders [24].

The authors propose that nitric oxide, K^+^ channels and protein synthesis may participate in the vasorelaxant effects of fenproporex, because such effects are attenuated in endothelium intact rat aortic rings by the application of L-NAME, TEA (a non-specific voltage-activated K^+^ channel blocker), and the combination of apamin and charybdotoxin or cycloheximide. Since glibenclamide did not modify vasorelaxant responses induced by fenproporex, K_ATP_ apparently is not involved in its vasorelaxant effects. It was concluded that fenproporex produces relaxant effects on phenylephrine-precontracted rat aortic rings, possibly by genomic mechanisms, the endothelial release of nitric oxide and/or stimulation of K_v_-, and to a lesser extent through SK_Ca_-, IK_Ca_- and BK_Ca_-channels [2].

## 4. Triiodothyronine

Thyroid hormones, prescribed to patients with thyroid gland dysfunction, can also be administered to obese euthyroid patients for weight loss. In several studies, the administration of thyroid hormone analogs to patients has led to a decrease in thyroid-stimulating hormone (TSH) and thyroxine (T_4_). However, the results are inconsistent with respect to weight loss [25].

Numerous experimental and clinical studies have demonstrated a relationship between thyroid hormones and the cardiovascular system [26]. There are various reports of a significantly modified cardiac function in patients with persistent subclinical thyroid dysfunction involving T_3_ and/or thyroxine (T_4_), which are thyroid hormones found in plasma and peripheral tissues, respectively. Triiodothyronine, the biological active thyroid hormone, is mostly generated by 5´-monodeiodination of T_4_ in peripheral tissues [27]. 

Even though the findings described in many publications are contradictory, triiodothyronine has been illegally sold and consumed as a nutritional supplement in several countries. In recent years, a series of Hong Kong-based case studies reported that several patients suffered intoxication from the illegal use of this thyroid hormone, with a fraction presenting thyrotoxicosis and thyrotoxic periodic paralysis [28]. Among other adverse events are anxiety, hyperthyroidism, insomnia, diarrhea and palpitations [25].

An increase in the basal metabolic rate is the main effect of thyroid hormones, which explains why hypothyroid patients tend to be overweight and hyperthyroid patients frequently undergo weight loss [14]. Whereas most effects caused by thyroid hormones in bone and heart tissue are due to their interaction with the alpha isoform of thyroid receptors, the effects in the liver (e.g., a reduction in lipids) are mediated mainly by the β isoform of these receptors. Furthermore, the indiscriminate use of thyroid hormone analogs accelerates the loss of bone mass and can lead to arrythmias and cardiac hypertrophy if taken for prolonged periods of time [28,29].

Recent studies have focused on the cardiovascular effects of thyroid hormones, such as one that showed an acute concentration-dependent relaxant effect of triiodothyronine on vascular smooth muscle. This hormone generated a greater relaxant effect on aortic rings with than without endothelium. Its relaxant effect was partially inhibited in the absence of endothelium and in the presence of L-NAME, TEA, atropine (a competitive nonselective antagonist at central and peripheral muscarinic acetylcholine receptors), ODQ, KT 5823, and the combination of apamin and charybdotoxin or cycloheximide. The authors suggest the possible participation of the NO-cGMP-PKG pathway and/or Ca^2+^-activated K^+^ channels (via activation of muscarinic receptors) in the observed vasorelaxant effect [27].

## 5. Amfepramone

Amfepramone, also known as diethylpropion, is a sympathetic mimetic amine similar to amphetamines that stimulates the central nervous system and increases blood pressure. It is an appetite suppressing drug used for the treatment of obesity [7,22,30,31,32]. Monotherapy with this drug demonstrated greater efficacy than the placebo [23].

Amfepramone is rapidly and almost completely absorbed after oral administration. It undergoes significant metabolism in the liver, producing many active metabolites. Twelve non-acidic metabolites were identified, formed from three key reactions: N dealkylation, reduced ketone function, and benzene ring hydroxylation. The metabolites are conjugated upon the formation of hippuric acid and mandelic acid. Ninety percent of the absorbed product, including the metabolites, is eliminated in the urine within 30–40 h, at least 5% in the unmetabolized form. The rate of urinary excretion of the drug is a function of pH, decreasing with alkaline urine. The excretion half-life of the amfepramone-metabolite complex is approximately 10.4 h. In acidic urine, this parameter is diminished to 1.5–3 h. The plasma half-life of amfepramone is approximately two hours [33].

Among its adverse events are transitory ischemic attacks, pulmonary artery hypertension, [23] vomiting, anxiety, headaches, euphoria, insomnia, leukopenia and agranulocytosis [19]. It can cross the blood-brain barrier as well as enter the placenta and is contraindicated for hyperthyroidism, severe hypertension and glaucoma [7,11,22,23].

According to some in vitro studies, amfepramone causes vasodilatation in endothelium-intact rat aortic rings pre-contracted with phenylephrine, an effect not found after the mechanical removal of this tissue. Hence, the vasorelaxant effect of amfepramone is apparently endothelium-dependent. Overall, the evidence suggests that amfepramone may produce direct relaxation in systemic vasculature. However, its predominant effect following administration by a systemic and central route seems to be vasoconstriction, possibly due to the activation of central mechanisms. 

The direct vasorelaxant effects on aortic rings generated by amfepramone reinforce previous findings from experiments carried out on dogs, in which the intravenous injection of amfepramone produced a dose-dependent depressor reaction [34] and a transient vasodepressor effect [35]. In contrast to these results are additional observations made in the same report. The intracerebroventricular administration of amfepramone in dogs elicited a marked pressor response [34] and the transient vasodepressor effect induced by the intravenous injection of amfepramone in dogs was followed by a vasopressor effect [35]. Moreover, vasorelaxant responses to the application of amfepramone in aortic rings do not coincide with one clinical study in which oral intake of amfepramone triggered ischemic attacks in the brain of an obese patient [36].

In the experiments conducted by Lopez-Canales et al. [31], the vasorelaxant response in rat aortic rings caused by amfepramone was unaffected by 4-AP and glibenclamide, while being significantly attenuated by L-NAME and TEA. This suggests the involvement of nitric oxide released by endothelium tissue and the activation of K^+^ channels by Ca^2+^, also supported by the fact that the mechanical removal of endothelium tissue blocked vasorelaxation. Additionally, the NO pathway and K^+^ channels have been implicated in the endothelial-mediated control of vascular tone. Nevertheless, the vasorelaxation prompted by amfepramone was unaffected by indomethacin and by clotrimazole, excluding the participation of prostacyclins, the endothelium-derived hyperpolarizing factor in the endothelium-mediated vasodilation.

In summary, the data suggest that vasorelaxant responses produced on phenylephrine-precontracted rat aortic rings by amfepramone involve the activation of endothelial nitric oxide synthase and the opening of Ca^2+^-activated K^+^ channels [31].

## 6. Final Considerations

Due to the technological advances and lifestyle of the Western world, obesity has been on the rise for decades [1,14]. Although diet and exercise are the most important factors for achieving weight loss, for certain people this is either not enough or not possible. As a result, a wide variety of drugs have been developed to help with weight loss, each one with a different therapeutic target. Whereas some evoke satiety, others suppress the appetite or burn fat at an accelerated rate [14].

In general, anorexigenics have been related over the years to certain adverse effects, regardless of their family or type. Due to their vasoconstrictive mechanism of action, cardiovascular problems are among such effects [1,3,5,37]. That is, these drugs lead to elevated levels of catecholamines in the blood, which causes a rise in blood pressure. Since the risk factors faced by obese patients are aggravated, anorexigenics are contraindicated for the management of obese patients [1,37].

According to the findings of the present study, on the other hand, fenproporex [2], amfepramone [31], clobenzorex [17] and T_3_ [27] administered under certain conditions do not increase blood pressure, but instead generate endothelium-dependent vasodilation via nitric oxide, potassium and voltage-dependent calcium channels. Consequently, these drugs could probably be used without adverse cardiovascular effects [2,17,27,31]. Further research is needed to explore the possible involvement of type M1 muscarinic receptors in the mechanism of action of the aforementioned drugs, a mechanism suggested in previous reports. In addition to clarifying their mechanisms of action, evaluation should be made of the potential of fenproporex, amfepramone, clobenzorex and T_3_ as treatments for obesity, especially in patients suffering from comorbidities such as hypertension. 

## 7. Conclusions

Although it is well-recognized that exercise and diet are the key factors in managing obesity or overweight, pharmacological treatments clearly play an important role under some circumstances. Some researchers have made the effort to find other pharmacological effects of anorectics distinct from the ones classically understood. These drugs have been contraindicated for obese and overweight patients due to their adverse effects, especially cardiovascular effects. Taking into consideration the vasodilator effect produced at the vascular-endothelial level by various anorectics, particularly fenproporex, amfepramone, clobenzorex and T_3_, they could potentially gain a safer profile for use in obese patients with cardiovascular disease.
